# Skin Viral Infections: Host Antiviral Innate Immunity and Viral Immune Evasion

**DOI:** 10.3389/fimmu.2020.593901

**Published:** 2020-11-06

**Authors:** Vivian Lei, Amy J. Petty, Amber R. Atwater, Sarah A. Wolfe, Amanda S. MacLeod

**Affiliations:** ^1^ Department of Dermatology, Duke University, Durham, NC, United States; ^2^ School of Medicine, Duke University, Durham, NC, United States; ^3^ Department of Immunology, Duke University, Durham, NC, United States; ^4^ Pinnell Center for Investigative Dermatology, Duke University, Durham, NC, United States; ^5^ Department of Molecular Genetics and Microbiology, Duke University, Durham, NC, United States

**Keywords:** cutaneous innate immunity, skin viruses, antiviral proteins, skin antiviral response, cutaneous microbiome, skin aging

## Abstract

The skin is an active immune organ that functions as the first and largest site of defense to the outside environment. Serving as the primary interface between host and pathogen, the skin’s early immune responses to viral invaders often determine the course and severity of infection. We review the current literature pertaining to the mechanisms of cutaneous viral invasion for classical skin-tropic, oncogenic, and vector-borne skin viruses. We discuss the skin’s evolved mechanisms for innate immune viral defense against these invading pathogens, as well as unique strategies utilized by the viruses to escape immune detection. We additionally explore the roles that demographic and environmental factors, such as age, biological sex, and the cutaneous microbiome, play in altering the host immune response to viral threats.

## Introduction

The skin is a dynamic barrier organ that establishes a clear boundary between the host and the outside world. As an immune organ, the skin actively surveils the surrounding environment and establishes an appropriate barrier and immune response to commensal microbiota including bacteria, fungi, and viruses. However, upon disruption of the skin barrier, the skin must orchestrate complex immune signals to protect against infiltration and attack by pathogenic invaders. Importantly, responses by the cutaneous innate immune system and its effectors play essential roles in early destruction of pathogens as well as establishment of an immune barrier to prevent systemic infection. This is accomplished *via* phagocytic cells (*i.e.* macrophages, neutrophils, and dendritic cells), leukocytes (*i.e.* natural killer (NK) cells, mast cells, basophils, and eosinophils), as well as epidermal keratinocytes. The introduction of pathogens activates these innate immune cells’ pathogen recognition receptors (PRRs), including toll-like receptors (TLRs), nucleotide-binding oligomerization domain (NOD)-like receptors, retinoic acid-inducible gene 1 (RIG-I)-like helicase receptors, and c-type lectin receptors. PRRs recognize different pathogen-associated molecular patterns (PAMPs) on microbes and damage-associated molecular patterns (DAMPs) that arise from damaged host cells, which subsequently leads to the induction of pro-inflammatory cytokines, such as tumor necrosis factor (TNF)-*α* and interferon (IFN)-*γ*, as well as chemokines that recruit phagocytic cells. Keratinocytes and infiltrating immune cells further the hostile environment to pathogens by generating peptides and proteins with distinct antibacterial, antifungal, antiviral capabilities (1).

**Figure 1:**
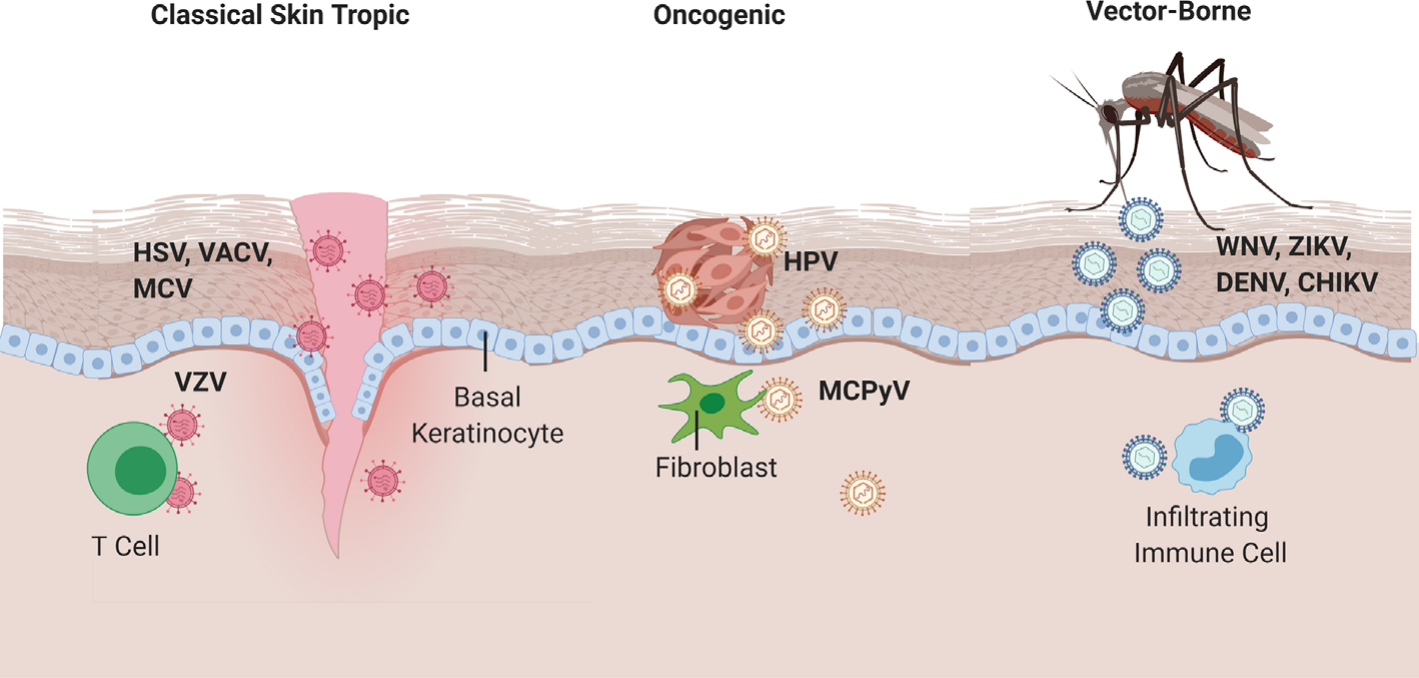
Viral entry of classical skin tropic, oncogenic, and vector-borne viruses. Classical skin tropic viruses such as herpes simplex virus (HSV), vaccinia virus (VACV), molluscum contagiosum virus (MCV), and varicella zoster virus (VZV) have tropism to skin epidermis where keratinocytes are the predominant cell type. HSV and MCV can enter the skin *via* defects in the skin barrier, which provide viruses with direct contact to the basal epidermal layers. VACV is introduced iatrogenically *via* vaccination needles. VZV inoculation occurs in the respiratory epithelia and hematogenously spreads to epidermis *via* infected T cells. Oncogenic viruses such as human papillomaviruses (HPV) and merkel cell polyomavirus (MCPyV) commonly take on their neoplastic potential in immunocompromised patients where the barrier to overcome immune defenses are significantly lower. HPV enters *via* micro-lesions and replicates in keratinocytes, whereas MCPyV has proclivities toward replication in dermal fibroblasts and CD4^+^ T cells, respectively. West Nile, Zika, Dengue, and Chikungunya viruses are introduced into the skin *via* mosquito vectors and cause a local inflammatory response that homes immune cells to the skin infection site, which allows for subsequent infection of migratory immune cells and potential for systemic spread.

Cutaneous viral infection presents a unique challenge to the skin’s immune system, as viruses have the ability to hijack host machinery to advance viral replication. As such, early abrogation of viral pathogenicity by the innate immune response establishes a protective antiviral state and limits the potential for systemic spread. Here, we provide an overview of viral entry mechanisms by various viruses with differing infection propensities, *i.e.* classically skin-tropic and oncogenic skin viruses, as well as vector-introduced skin viruses. We review how these viruses uniquely interact with different aspects of the cutaneous innate immune system, and we further explore some evolved viral mechanisms that directly interfere with the host innate immune response. Lastly, we provide insights on how demographic and environmental factors, such as host age, biological sex, and the commensal microbiome, contribute to various aspects of innate antiviral immunity in the skin (**Figure 1**, **Table 1**).

**Table 1:** Summary of cutaneous viruses, their cell tropism, their innate immune sensors and evasion targets, and populations vulnerable to viral infection.

Virus	Cell Tropism	Innate Immune Sensors	Immune Evasion Targets	Vulnerable Populations
**Classical Skin-Tropic Viruses**				
Herpes Simplex Virus (HSV)	Epidermal keratinocytes	Toll-like Receptors (TLR): TLR2, TLR3, TLR9 NOD-like receptors Melanoma differentiation-associated gene 5 (MDA5) Interferon-inducible protein 16 (IFI16) Helicases: Ku70, DHX9, DHX36, DDX60	Macrophage receptor with collagenous structure (MARCO) Cyclic guanosin monophosphate synthase (cGAS)/Stimulator of interferon genes (STING)-mediated interferon production Absent in melanoma 2 (AIM2)	Neonatal Immunocompromised Males – HSV-1 Females – HSV-2
Vaccinia Virus (VACV)	Dendritic cells, macrophages, monocytes	Not yet identified	Not yet identified	Patients with history of atopic dermatitis
Molluscum Contagiosum Virus (MCV)	Epidermal keratinocytes	TLR3, TLR9	Dermal immune cells	Children
Varicella Zoster Virus (VZV)	Primary infection at upper respiratory epithelium, hematologic transport to skin keratinocytes *via* infected T cells	TLR9 cGAS	Not yet identified	Children Elderly (Herpes Zoster) Immunocompromised Males
**Oncogenic Viruses**				
Human Papillomavirus (HPV)	Basal keratinocytes	TLR3, TLR7, TLR8, TLR9 AIM2 Interferon-gamma inducible protein 16 (IFI16)	Interferon regulatory transcription factors: IRF1, IRF3 C-C motif chemokine ligand 20 (CCL20)	Immunocompromised
Merkel Cell Polyomavirus (MCPyV)	Keratinocytes, dermal fibroblasts	TLR9	Nuclear factor-γB Major histocompatibility complex class I (MHC-I)	Immunocompromised Females
**Vector-borne Viruses**				
*Flaviviruses –* Zika (ZIKV), West Nile (WNV),	Dendritic cells – ZIKV, DENV	TLR3	Pathogen recognition receptors – WNV	Females – DENV
Dengue (DENV)	Keratinocytes – WNV	MDA5	Nuclear factor-κB – ZIKV	Elderly – WNV
		Retinoic acid-inducible gene I (RIG-I)		
Chikungunya Virus (CHIKV)	Dermal fibroblasts			

## Classical Skin-Tropic Viruses

### Herpes Simplex Virus (HSV)

Herpes simplex virus (HSV) type-1 and 2, of the *Herpesviridae* family, are enveloped double-stranded DNA viruses that are notable for their neurotropism to the dorsal root ganglia and trigeminal ganglia after primary infection at a mucocutaneous site (2). Primary and reactivated infections are marked by tender grouped erythematous vesicles with varying presentations and degrees of severity (3). HSV-1 is typically characterized by oro-facial lesions with primary infection most often occurring in childhood, whereas HSV-2 is traditionally known as a sexually transmitted infection producing genital lesions, although both types can be found at either site (4). In immunocompromised and neonatal patients, HSV has the potential to disseminate and cause severe morbidity and mortality (3).

In both primary and reactivated infections, viral entry and replication largely occur in the epidermis, where keratinocytes are the predominant cell type. Host cell entry is coordinated by seven HSV glycoproteins; however, four glycoproteins (gB, gD, gH, and gL) are necessary and sufficient for complete viral fusion (5). Viral entry steps start with initial attachment to heparan sulfate proteoglycans (HSPGs) on keratinocytes *via* gB and gC. Subsequent fusion of the viral envelope with the plasma membrane is mediated by gB and heterodimer gH/gL (6, 7). Envelope glycoprotein gD additionally interacts with cell surface receptors nectin-1, nectin-2, and herpesvirus entry mediator (HVEM) to aid in viral envelope fusion with the plasma membrane (8, 9). After fusion, HSV viral spread relies on the trans-Golgi network for delivery of viral glycoproteins and particles with resultant infection of nearby cells *via* cell–cell junctions (10, 11).

At the cell surface, Toll-like receptor (TLR) 2 senses viral gB and gH/gL and activates the nuclear factor *κ*B (NF-*κ*B) pathway to induce expression of pro-inflammatory cytokines (*e.g.* tumor necrosis factor (TNF)-*α*, interleukin (IL)-6 and IL-12) and chemokines (*e.g.* CC chemokine ligand 2 (CCL2)) (12–15). Once within the cell, HSV nucleic acids activate TLR3 and TLR9 in the endosomes, while a slew of PRRs (*i.e.* NOD-like receptors, melanoma differentiation-associated gene 5 (MDA5), interferon-inducible protein 16 (IFI16), and several helicases (Ku70, DHX9, DHX36, DDX60)) sense HSV DNA and RNA in the cytoplasm (16). Together, PRR activation confers type I and III interferon signaling in both human keratinocytes and infiltrating monocyte-lineage cells (17–21). Several induced interferon stimulated gene (ISG) products, such as myxovirus (Mx) A and double-stranded RNA-activated protein kinase (PKR), have direct antiviral properties against HSV, such as limiting viral replication and initiating autophagy to limit cell–cell spread (22). The importance of these many facets of the innate immune antiviral response are highlighted in observations that patients with tyrosine kinase 2 (TYK2) deficiency, who have impaired type I IFN, IL-6, and IL-12 responses, have increased frequency of recurrent HSV infections (23).

Additional innate host defense regulators acting prior to the canonical IFN signaling pathways have also been discovered to play roles in the battle against HSV. For example, promyelocytic leukemia nuclear bodies associate with histone chaperones to capture viral DNA and block HSV replication (24, 25). Keratinocytes were also found to release IL-1*α* and IL-36 to bolster the antiviral state by acting as early alarm signals for leukocyte recruitment and increasing cellular sensitivity to type I IFN signaling, respectively (26, 27).

Recent discoveries have also identified novel potential roles of NK cells to contribute directly to innate protection against HSV infection. A 2003 study in mice identified that NK cells provided a critical source of early IFNs to control HSV-2 infection and that mice deficient in NK cells had enhanced susceptibility to HSV (28). Corroborating these observations is a case report in 2004 of two individuals with NK cell deficiency who were observed to have severe disseminated HSV-2 infection (29). Absence of NK cells resulted in a diminished CD4^+^ and CD8^+^ T cell responses, and the presence of NK cells alone were identified to be able to rescue dysmorphic CD8^+^ T cells to mount an effective CD8^+^ T cell response even in the absence of CD4^+^ T helper cells (30). These findings propose a potential role of NK cells to mediate and bridge innate and adaptive immune responses. Further investigations can be conducted to elucidate the specific mechanisms utilized by NK cells to enhance T cell responses and determine whether NK cells exposed to HSV confer a ‘memory’ response to more readily bolster both innate and adaptive immune functions upon HSV reactivation. These discoveries may present NK cells as attractive targets to enhance both arms of the immune response against HSV infection. The role of innate lymphoid cells (ILCs) has been additionally studied in the context of HSV infection, though *in vivo* mouse studies showed that ILC-deficiency showed no difference in survival or disease severity (31).

Despite the many innate immune players against HSV, the virus has evolved mechanisms to usurp host machinery and enhance infectivity. For example, HSV was discovered to use scavenger receptors to increase affinity of surface protein interactions (32), inhibit intracellular viral DNA sensing (33, 34), dampen pro-inflammatory cytokine production and inflammasome formation (35), and directly abrogate type I IFN signaling (36). These mechanisms have rendered HSV to be one of the most successful viruses capable of infecting other cell types, including fibroblasts, lymphocytes, and leukocytes (8). Unsurprisingly, HSV’s ability to counteract multiple facets of the early, innate cutaneous immune response helps to explain its capacity to successfully infect beyond the initial infection site and cause latent disease. Given the plethora of studies of viral mechanisms and viral targets for immune evasion, HSV is primed as a viable target to study ways to strengthen innate antiviral immune responses, both IFN-dependent and IFN-independent, to provide different avenues of attenuating disease severity.

### Vaccinia Virus

Vaccinia viruses are large, enveloped double-stranded DNA viruses of the *Poxviridae* family. Due to highly conserved structural proteins across orthopoxviruses, VACV is often used to immunize against smallpox caused by variola virus (37). All human orthopoxvirus infections are zoonoses and typically present as localized or disseminated papules, vesicles, or scabs that may be accompanied by fever, lymphadenopathy, malaise, and myalgia (38).

VACV replication preferentially occurs in cutaneous sites with compromised barrier function (39), where there is increased access to the basolateral membrane (40). Viral entry begins with attachment of four viral proteins (A26, A27, D8 and H3) of the mature virion to cell surface glycosaminoglycans (GAGs), extracellular matrix proteins, and, at lipid rafts, integrin membrane receptors (41, 42). Following attachment is an intricate synchrony of twelve entry proteins that compose the fusion complex, which introduces viral DNA into the cell (reviewed in 43).

Infection with VACV is uncommon when exposure occurs in a healthy cutaneous environment where innate immune responses effectively suppress viral pathogenicity. In fact, a study by Rice and colleagues showed that enhancement of early pro-inflammatory signals using a scarification model of viral delivery significantly decreased lethality of VACV. The group proposed that scarification allowed keratinocytes to actively produce an antiviral state through secretion of chemokines and cytokines (44). These findings are corroborated by discoveries that TNF-receptor knockout and IL-1 receptor type 1 knockout mice had larger cutaneous lesions and higher viral copies compared to their wild type counterparts (45, 46). *In vitro*, VACV viral infection of epidermal Langerhans cells (LC) and plasmacytoid dendritic cells (pDCs) resulted in inhibition of their ability to elicit cytokine production, including IFN-*α* and IFN-*γ* (47, 48). Activated NK cells also secrete necessary IFN-*γ* to attenuate early infection and promote VACV clearance (49–51). Together, these findings suggest a key role in early innate immune signaling in preventing viral lethality; these signals are essential for VACV vaccine efficacy.

Though typically regarded as safe, VACV vaccination has the potential to cause eczema vaccinatum or progressive vaccinia, both severe and potentially lethal complications (52, 53). Occurring mostly in individuals with a history of atopic dermatitis (AD), a disease that is distinguished by barrier defects resulting from disrupted terminal epidermal differentiation, disseminated VACV includes a generalized vesiculopustular eruption that can progress to large non-healing lesions and predispose individuals to sepsis (54, 55). Viral progression is theorized to be due to reduced capability of AD skin’s innate immune mechanisms to subvert viral attack. With VACV’s preferential infection of dendritic cells, macrophages, and monocytes (56), infection of epidermal antigen-presenting LCs at the early stage impairs release of pro-inflammatory cytokines and IFNs (48). Next, attempts to limit viral spread *via* programmed cell death are offset by AD skin’s hyper-proliferative state, which presents the virus with many new targets (57). Moreover, the skew towards Th2 responses in AD, with increased IL-4 and IL-13 expression in particular, further decreases antiviral cytokines and type I and II IFNs (58, 59). Consequently, this results in reduced expression of antimicrobial proteins such as human *β*-defensin (hBD) 3 and human cathelicidin LL-37, which have been shown to directly deter VACV pathogenicity (60, 61). Together, the compromised immune landscape in AD skin provides fertile ground for VACV spread. Given the strong association between VACV (and also HSV) dissemination and AD, future studies are warranted regarding how alterations in terminal epidermal differentiation affect innate antiviral immune signatures at homeostasis as well as upon viral challenge.

### Molluscum Contagiosum Virus

Molluscum contagiosum virus, an enveloped linear double-stranded DNA virus of the *Poxviridae* family, is introduced *via* direct contact with infected skin or fomites (62). Although MCV infection is common, specific studies on viral entry mechanisms have been limited due to lack of working *in vivo* and *in vitro* models. Early electron microscopy of MCV showed preferential infection of keratinocytes in the basal layers at the outset of primary infection (63, 64). Similar to other viruses with tropism to the basal layer, micro-abrasions in the skin provide MCV a direct pathway of entry, and it has been well documented that individuals with skin barrier defects have increased susceptibility (65, 66). Viral proliferation then continues in mitotically active keratinocytes and expands apically, giving rise to distinct dome-shaped papules called molluscum bodies. Viral dissemination occurs as viral particles exit *via* a keratinized tunnel at the umbilicated center of the lesion (67).

MCV is notable for its ability to evade immune detection as it replicates within epidermal keratinocytes; it forms enclosed molluscum bodies that effectively evade dermal immune detection (68, 69). Interestingly, reports that physical manipulation of molluscum bodies results in local inflammation and ultimate resolution of the infection posit the notion of viral clearance by nearby dermal immune cells (70, 71). Although studies of specific innate immune responses to MCV are limited, one study suggests that MCV activates TLR3 and TLR9 in epidermal keratinocytes. They additionally observed upregulation of IFN-*β* and TNF-*α* in the environment surrounding molluscum bodies (72). Work by Vermi et al. further identified plasmacytoid and type I IFN-induced dendritic cells as key effectors in spontaneous regression of MCV in the aforementioned inflammatory setting (73). While MCV’s preference toward epidermal replication allows it to escape dermal immune detection, it remains unclear whether and how epidermal Langerhans cells contribute to immune responses to MCV infection and whether MCV has evolved mechanisms to silence LC contributions to immune surveillance.

### Varicella Zoster Virus

Varicella zoster virus is another neurotropic enveloped, double-stranded DNA virus of the *Herpesviridae* family with primary infection consisting of a generalized pruritic vesicular eruption along with fever, headache and malaise (74). Unlike the previously discussed skin-tropic viruses, infection of epidermal keratinocytes is introduced *via* hematologic transport of infected T cells after primary inoculation in the upper respiratory epithelium (75, 76). VZV utilizes gB and heterodimer gH/gL, conserved fusion machinery of herpesviruses, for attachment and entry into keratinocytes. Within the skin, cell–cell fusion generates multinucleated infected cells that reside within the vesicular skin lesions. Studies show that VZV fusion protein gB possesses components on both its ecto- and cytoplasmic domains that are essential for infectivity: gB drives VZV’s replication, cell–cell fusion, and characteristic syncytial formation (77, 78). However, additional studies suggest that VZV virulence requires careful regulation of gB, as gain-of-function mutations in gB have been shown to limit viral spread in human skin (79).

Given the poor outcomes in VZV-infected individuals with adaptive immune deficiencies, early establishment of an antiviral state in the skin is vital. These responses work effectively to limit disease severity and activate cell-mediated immunity. Cytosolic sensing activates stimulator of interferon genes (STING)-mediated IFN-*γ* production to upregulate antiviral genes, like *MxA* and *OAS*. TLR9 dependent sensing of VZV is also noted to trigger massive IFN-*α* release by pDCs (80, 81). Exogenous treatment with IFN-*α* has been shown to abrogate VZV severity through inhibition of viral replication *via* interferon regulator factor (IRF) protein 9 (82, 83). However, IFN-*α* signaling was not sufficient to completely terminate VZV transmission due to down-regulation of this pathway by viral gene products (75). Natural killer cells also prevent viral spread by killing infected cells, and their absence has been linked to severe infection (84, 85). Given the discoveries of the important role of NK cells during innate immune signaling and priming adaptive responses in other skin viruses, studies of the specific functions of NK cells in the context of VZV can provide promising avenues of discovery into establishment of an early antiviral state.

## Oncogenic Viruses

### Human Papillomavirus

Human papillomaviruses are non-enveloped double-stranded DNA viruses that can be transmitted through direct skin-to-skin contact (86). There are more than 200 described HPV types. The alpha HPVs (*i.e.* HPV16, 18, 31, 33, 35, 39, 45, 51, 52, 56, 58 and 59) are considered high risk or carcinogenic and have been identified as etiologic agents of a multitude of cancers, including cervical, oropharyngeal, vaginal, vulvar, penile, and anal cancers (87, 88). Beta and gamma types are considered possibly carcinogenic or non-carcinogenic. Several studies have identified potential contributory roles of beta HPVs to non-melanoma skin cancer when associated with ultraviolet radiation (89). The low risk non-carcinogenic HPVs are known to cause benign lesions such as anogenital, palmar, and plantar warts (90).

Viral penetration into the epidermis is facilitated *via* microlesions and HPV’s replication cycle starts at the mitotically active basal layer (91). Once within the basal layer, viruses gain entry into the cells through endocytosis, which are enabled by viral proteins L1 and L2 that help the virus interact with the cell surface. Molecules such as HSPGs and syndecan-1 are putative targets of HPV that enable viral trafficking into the host cell (92). After internalization, HPV virions reach the nucleus through the clathrin-mediated endocytic pathway (93, 94).

Within the basal layers, HPV DNA copy number is low and viral replication is slow. As viral replication speeds up and the virus leaves the basal layer to reach the upper layers of the epidermis, innate and adaptive immune responses become more important in surveilling and controlling viral spread (95). HPV DNA within a host cell is recognized by innate pathogen sensors, including absence in melanoma 2 (AIM2), interferon-gamma inducible protein 16 (IFI16), and cyclic guanosine monophosphate-adenosine monophosphate synthase (cGAS) (96–98). AIM2 inflammasome activation results in maturation of caspase-1 and IL-1*β* in HPV16-infected keratinocytes (99). TLR activation in keratinocytes by HPV also results in release of pro-inflammatory cytokines such as TNF-*α*, IL-8, C-X-C motif chemokine ligand 9 (CXCL9), and type I interferon (IFN-*α* and -*β*) (100). In fact, higher expression of TLRs was found to be correlative with clearance of initial HPV16 infection in women (101).

HPV-infected keratinocytes additionally recruit macrophages, Langerhans cells (LCs), natural killer (NK) cells, and T lymphocytes in the initial antiviral response. TLR activation in macrophages and LCs through NF-*γ*B and interferon response factor (IRF)-3 further promotes the release of TNF-*α*, IFN-*γ*, IL-1*β*, IL-12 and IL-18, which can in turn activate other inflammatory cells through paracrine signaling. IL-1 and TNF-*α* have also been shown to downregulate the transcription of viral oncoproteins E6 and E7 (100). Though there is limited evidence on the role NK cells play in controlling HPV infections, it was reported that patients with functional NK deficiencies were more susceptible to HPV infection and HPV-associated cancer (102). Together, these studies highlight the importance of host innate immunity during the initial antiviral responses against HPV in cutaneous tissues.

Many studies provide evidence that HPV has evolved mechanisms to counter host immune responses. HPV-infected cells can reprogram the local immune milieu to promote chronic inflammation and subsequently carcinogenesis. HPV E6 protein can directly target IRF3 while E7 protein interferes with the antiviral and pro-apoptotic functions of IRF1 *via* protein–protein interactions, leading to suppressed IFN signaling and downstream responses (103–105). Additionally, HPV infection was found to interfere with LC homeostasis due to the suppression of C-C motif chemokine ligand 20 (CCL20), a chemokine critical for the repopulation of CD1a^+^ LC precursor cells in the epidermis (106). It was shown that viral E7 protein abrogates the binding of CCAAT/enhancer-binding protein beta (C/EBP*β*) in the promoter region of CCL20. As a result, CCL20-directed migration of LCs and subsequent antigen-presentation in the epithelium is suppressed, allowing for viral persistence (106). In summary, HPV modulates several host cellular pathways to evade immune responses, leading to virus-mediated immunosuppression and neoplastic development. However, given the diversity of HPV types and their various neoplastic or benign propensities, further investigations are needed to identify differential mechanisms utilized by the host to respond to various HPV types, as well as how certain specific HPVs are able to subvert host immune signaling to impart immunosuppression and impart neoplastic potential.

### Merkel Cell Polyomavirus

Merkel cell polyomavirus belongs to the *Polyomaviridae* family which consists of non-enveloped, double-stranded DNA viruses that have infectious and tumorigenic potential (107). Since the initial identification in Merkel cell carcinoma (MCC) in 2008, many reports have provided additional evidence of the causal relationship between MCPyV and MCC (108–112). MCC is an aggressive cancer that is characterized by a rapidly expanding, asymptomatic, erythematous dome-shaped tumor that presents often on sun-exposed areas of the skin (113).

It remains under debate which cutaneous cell type(s) MCPyV primarily infects due to poor replication of MCPyV in *in vitro* cultures (114). Keratinocytes were thought to be the primary target due to chronic cutaneous shedding of MCPyV (115). However, a recent report showed that MCPyV preferentially infects human dermal fibroblasts (116). Viral attachment relies on recognition of sulfated GAGs and interaction with sialylated oligosaccharides containing the Neu5Ac*α*2-3Gal linear motif by viral capsid protein, VP1 (117, 118). MCPyV eventually enters target cells through caveolar/lipid raft-mediated endocytosis (119).

Many recent reports suggest the important role the host immune system plays in MCPyV infection and MCC development. First of all, immunocompromised patients are more likely to develop MCC (120). Secondly, high intratumoral CD8^+^ T cell counts and immune transcripts are associated with more favorable outcomes in MCC patients (121, 122). Innate immune responses were thought to play a critical role in the initial sensing and clearance of MCPyV virions. Shadzad et al. reported that TLR9, a critical sensor for viral and bacterial dsDNA, is downregulated by MCPyV large T antigen during infection (123). Additionally, MCPyV small T antigen negatively regulates NF-*γ*B-mediated inflammatory signaling by inhibiting IKK*α*/IKK*β*-induced I*γ*B phosphorylation, further dampening host antiviral responses (124). Lastly, MCPyV-positive MCC tumors were discovered to have lower expression of major histocompatibility complex class I (MHC-I) compared to MCPyV-negative MCC samples, suggesting another potential mechanism by which MCPyV-infection cells escape immune destruction (125). However, precise interactions between MCPyV and the host immune system are largely unknown. Further work is needed to elucidate the various mechanisms by which MCPyV subverts host immune surveillance to establish persistence.

## Vector-Borne Skin Viruses

Mosquitos infect hundreds of millions of people around the world annually, introducing individuals to pathogenic bacteria, parasites, and viruses that have the potential to cause severe systemic illness in the host and, with Zika virus, their offspring (126, 127). Despite the prevalence of mosquito-borne illnesses and their threat to global human health, little is known about the early stages of cutaneous infection.

Zika virus (ZIKV), West Nile virus (WNV), and Dengue virus (DENV) belong to the *Flaviviridae* family and are enveloped RNA viruses. ZIKV and DENV have a predisposition to infect cutaneous dendritic cells, whose migratory characteristic allows for rapid dissemination and viremia (128, 129). WNV has been shown to preferentially infect keratinocytes, though it is capable of infecting dendritic cells as well (130, 131). Flaviviral envelope (E) glycoprotein is key to initial viral entry *via* low-affinity attachment to GAGs on the target cell surface (132, 133). More specific attachment to a wide array of entry receptors that help facilitate internalization into dendritic cells has been identified, including C-type lectin receptors, *αvβ*3 integrins, T-cell immunoglobulin and mucin domain (TIM) and TYRO3, AXL and MER (TAM) receptors (134). Clathrin-dependent endocytosis then allows for viral fusion into the target cell (135).

Chikungunya virus (CHIKV) is also an enveloped RNA virus but belongs to the *Togaviridae* family with tropism to dermal fibroblasts (136). CHIKV viral glycoprotein E2 interaction with cell surface GAGs, TIM family receptors, and prohibitins has been shown to assist with early interactions of CHIKV with the target cell, although CHIKV is able to infect in the absence of these proteins (137). Similar to flaviviruses, CHIKV utilizes clathrin-dependent endocytosis to generate a low pH environment to cause conformational changes in glyocoprotein E1 and permit fusion (138).

Once in the skin, ZIKV, WNV, DENV, and CHIKV all trigger PRRs retinoic acid-inducible gene I (RIG-I), TLR3, and melanoma differentiation associated gene-5 (MDA-5). Next, pro-inflammatory cytokine and chemokine signaling is coupled with activation of IFN-*β* and antiviral proteins, including members of the OAS, Mx, interferon stimulated gene (ISG), and interferon induced proteins with tetratricopeptide repeats (IFIT) families, in keratinocytes and dermal myeloid cells (81, 129, 139–142). Specific to ZIKV, our group recently identified a novel IFN-independent pathway of antiviral protein induction *via* IL-27. Uniquely, signal transducer and activator of transcription (STAT) 1- and interferon regulatory factor (IRF) 3-dependent IL-27 signaling was able to induce antiviral proteins OAS1, OAS2, OASL2, and MX1 in keratinocytes and reduce ZIKV pathogenicity when the virus was introduced *via* a cutaneous, and not intravenous, route (143). These results suggest a potential avenue to distinctly upregulate cutaneous antiviral proteins independent of interferon signaling, although whether this pathway confers similar resistance to other vector-borne viruses remains to be discovered.

Given arboviruses’ predilection to infect immune cells, the recruitment of distal immune cells to the dermis may not be as advantageous to the host as is the case for many other pathogens. After an early infection in the epidermis, a second round occurs when the immune response homes arbovirus-susceptible monocytes and monocyte-derived dendritic cells to the site (144). These infected immune cells then travel to the draining lymph nodes to continue systemic spread. This begs the question of whether pathogenicity can be reduced or limited to the epidermis by dampening inflammatory signaling. One group observed a 75–90% reduction in infection of LCs, macrophages, and dermal dendritic cells when cytokine IL-1*β* expression was inhibited (128).

An additional non-viral factor also contributes to the immune picture. Intriguingly, mosquito saliva has been shown to significantly alter the early innate signatures to enhance viral spread. Mosquito saliva protein D7 inhibits DENV virions and envelope proteins (145). ZIKV-activated NF-*ĸ*B signaling is inhibited by salival protein LTRIN (146). In CHIKV, mosquito saliva suppresses Th1 cytokine (IFN-*γ* and IL-2), TLR3, and chemokine expression while simultaneously pushing toward a Th2 polarity—which, as we have discussed, is a less advantageous antiviral profile from the host perspective (147, 148). Decreases in expression of PRRs and antiviral proteins with specific targeting of flaviviruses (OAS1, MX1, and ISG20) were also observed in WNV-infected keratinocytes (149, 150).

The unique mode of inoculation of vector-borne viruses at the skin presents an alluring rationale to study potential methods of undermining viral pathogenicity when the infection is still local and while innate immune responses predominate. However, the frequency of mosquito bites and the lack of urgency to seek medical attention prior to systemic infection may pose a conceivable difficulty for translation into clinical practice.

## Demographic and Environmental Contributors To Host Antiviral Responses

**Figure 2:**
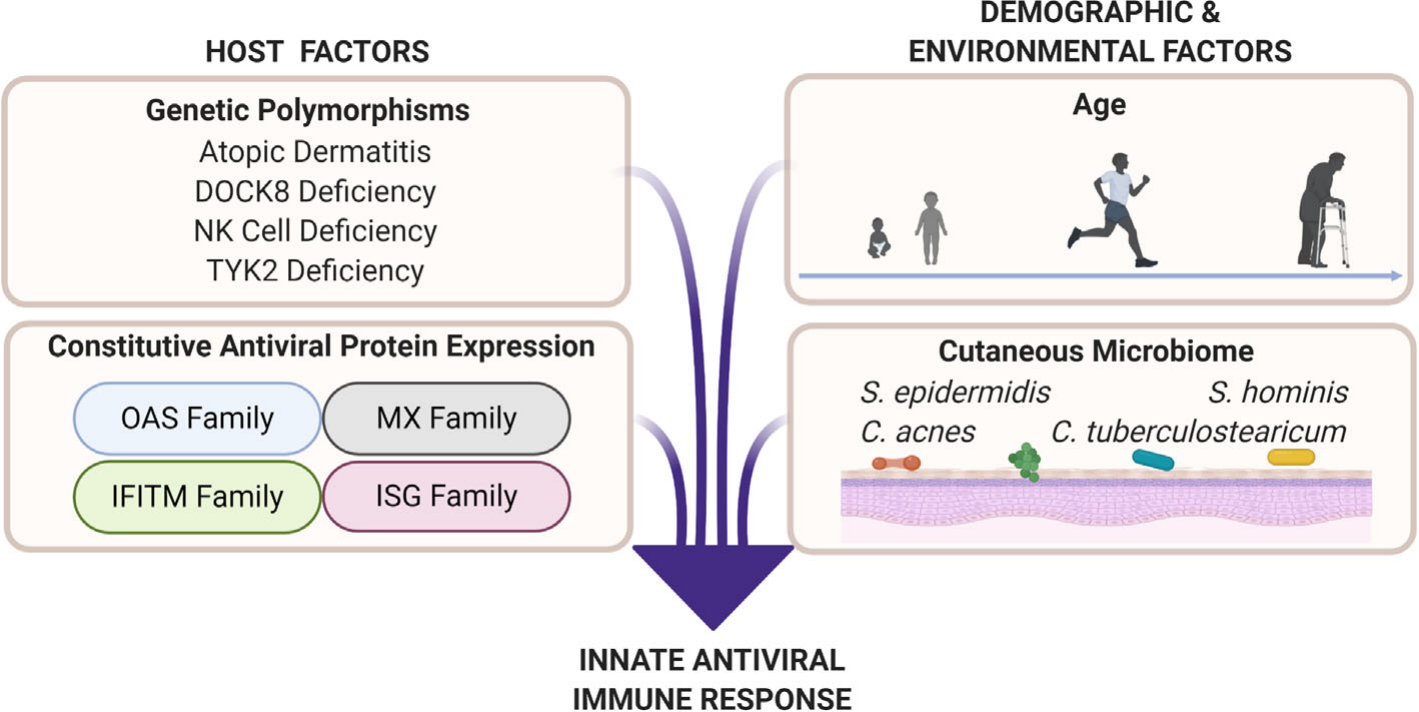
Cutaneous antiviral immune responses are influenced by host as well as demographic and environmental factors. Genetic polymorphisms that result in atopic dermatitis, dedicator of cytokinesis 8 (DOCK8) deficiency, natural killer (NK) cell deficiency, and tyrosine kinase 2 (TYK2) deficiency produce unique immune profiles that are disadvantageous for viral protection. Professional antiviral proteins such as those in the oligoadenylate synthetase (OAS), myxovirus resistance (MX), interferon-induced transmembrane (IFITM), and interferon-stimulated gene (ISG) families are part of the innate antiviral response. These proteins exert their antiviral abilities by inhibiting various parts of the viral replication cycle (151). Factors such as age (see **Figure 3**), biological sex, and cutaneous microbiome have potential to deter or enhance innate antiviral responses. Microbial interactions, such as bacteria–viral, viral–viral, and fungal–viral, can possibly produce antiviral effectors or influence host antiviral responses.

As a frontline organ of defense against the outside world, maintaining integrity of the skin barrier and function is critical to the organ’s success in combating potential invaders. However, increasing studies show that, like other regenerating organs, the skin is constantly adapting in response to a multitude of environmental factors (**Figure 2**).

### Age

**Figure 3:**
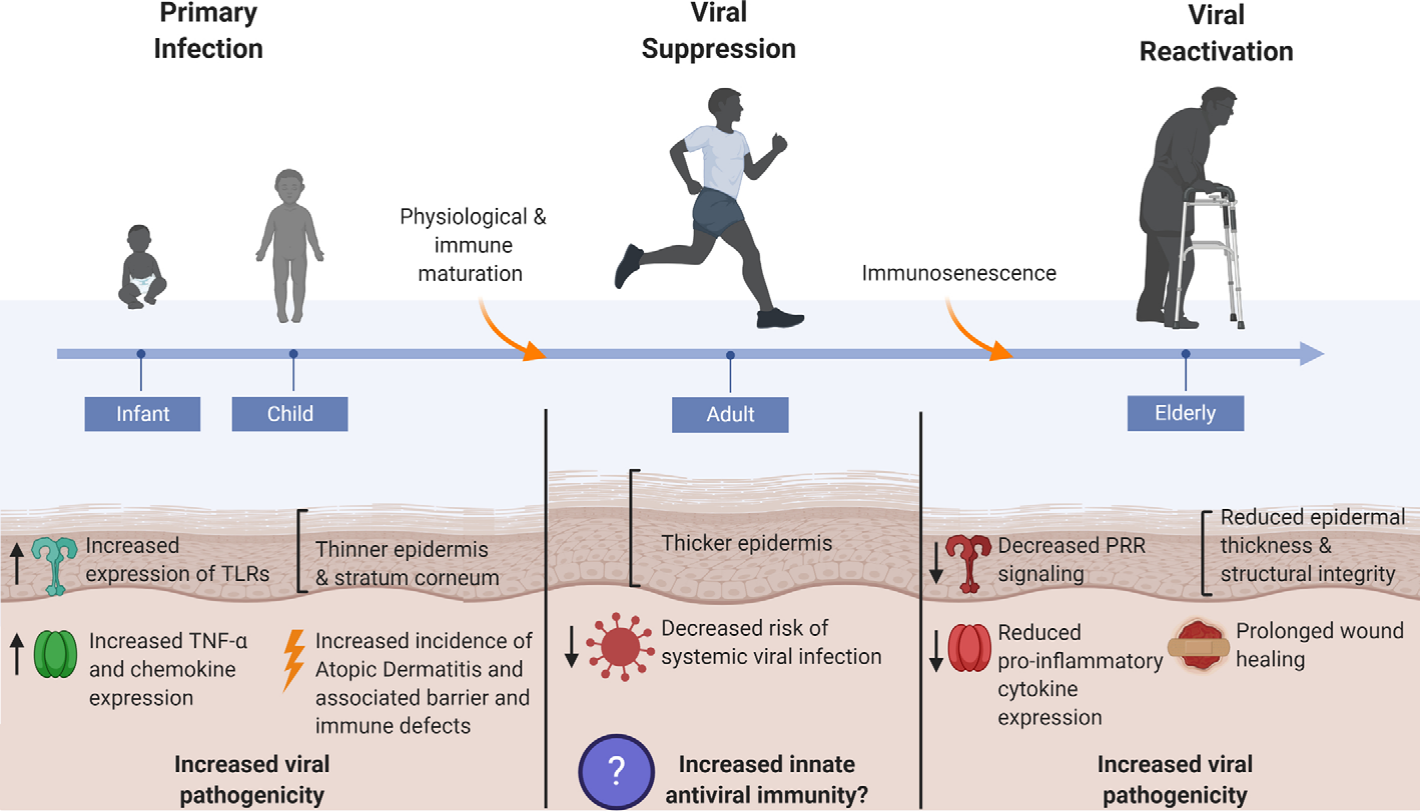
Skin’s antiviral protection changes throughout age. Systemic viral infections are most prevalent at the young and elderly ages where factors such as epidermal thickness and cutaneous innate immunity are markedly different from healthy adult human skin. Thin skin leads to increased susceptibility to micro-injuries and abrasions, thereby providing direct avenues for viral entry. Dysregulated innate immune signaling, consequent to immunological immaturity or immunosenescence in the young and elderly, respectively, furthers the risk of systemic viral infection as immune defenses cannot adequately control early viral propagation. The young and elderly are also at increased risk for viral pathogen exposure due to compromises in skin barrier integrity that manifest in the form of atopic dermatitis in the young and chronic non-healing wounds in the elderly.

Given the skin’s constant contact with potential pathogens, the susceptibility of certain patient populations to skin viruses is an interesting area of investigation. Notably, age appears to play a role in the host immune defenses against viral invaders (**Figure 3**). Some trends are more obvious: MCV and VACV show increased incidence and more severe effects in children as prevalence of AD is highest in this age group, and as previously discussed, the AD milieu contributes to increased viral pathogenicity and impaired antiviral responses (152–154). However, there is less clarity on why certain age groups are more afflicted with other cutaneous viral infections. Intriguingly, prenatal, neonatal, and elderly populations have demonstrated increased susceptibility to systemic malaise and higher risk of mortality compared to young and mature adults. For example, whereas only mild symptoms would typically result from primary HSV infection in children and adults, preterm and neonatal infants, if untreated, only have a 40% chance of survival (155). Similarly, in the elderly population, reports have emerged suggesting that HSV increases the risk for development of neurological diseases like Alzheimer’s and may be a direct infectious etiology (156). Moreover, while VZV dissemination occurs most commonly in children due to primary infection, suppression of the virus is maintained throughout adulthood. However, reactivation, which only occurs after VZV overthrows immune safeguards and presents in the form of herpes zoster, occurs most frequently in elderly individuals or upon immunosuppresion (157).

One potential explanation for these observations is alteration in the skin’s physical barrier with aging. Preterm and neonatal infants have a thinner epidermis and stratum corneum, and a similar observation applies to the elderly population where cutaneous structural integrity deteriorates and skin thickness is once again reduced (158, 159). Such changes to skin integrity may render it more susceptible to micro-injuries and therefore subsequent pathogen exposure and infection. Functional studies on whether the rate of viral infectivity is enhanced in the setting of thin, fragile skin barriers are limited. Theoretically, decreased epidermal thickness may provide for earlier access to deeper skin layers, which could potentially lessen the time the virus spends replicating at the initial infection site prior to systemic spread, and therefore limit the time available for propagation of early innate immune responses as well as initiation of adaptive immune responses. Additional concerns are warranted in the elderly where the skin’s wound healing capabilities are also reduced, thereby allowing for increased pathogen exposure (159).

Age also has profound effects on certain aspects of the skin’s innate antiviral defenses. For example, in WNV infection, which usually afflicts individuals >60 years of age, worse outcomes were identified in mice with dysregulated TLR7 and STING signaling, both with critical roles in initiating antiviral signaling cascades (160, 161). Generally, older individuals exemplified decreased PRR signaling and decreased induction of pro-inflammatory cytokines and chemokines in several cutaneous compartments, including sebaceous glands, sweat glands, and epidermis (162). Surprisingly, prenatal skin actually exhibited higher levels of TLRs (1–5) compared to adults, and neonatal keratinocytes demonstrated greater secretion of TNF-*α* and several chemokines when stimulated with poly (I:C), a synthetic dsRNA used to mimic viral nucleic acids (163). It is unclear how this dichotomy corresponds to viral preference and susceptibility at different age groups, although similar outcomes of greater morbidity and mortality in both age groups highlight the importance of better understanding the regulators and effectors of innate antiviral immunity.

Studies have additionally identified discrepancies in the expression levels of cutaneous antimicrobial peptides and proteins at the extremes of age. For example, neonatal skin was observed to express increased levels of antimicrobial peptides LL-37 and hBD2 compared to adults in both mice and humans (164). Contrastingly, reduced levels of antimicrobial peptides were observed in aged skin compared to adult skin (165). While these studies begin to point toward differing antimicrobial signatures across age groups, investigations specifically looking at antiviral proteins and their functional implications are currently lacking.

### Biological Sex

Biological sex poses another important variable when considering immune defenses against viral pathogens. Sex differences in innate and adaptive immunity have been well characterized in humans; known to us is that infant and adult males mount weaker innate and adaptive immune responses to pathogens compared to females and are, therefore, theorized to be more susceptible to viral infections. Particularly in the context of innate immunity, varied responses to pathogens can be explained by differential expression in TLR and type I IFN signaling between sexes, wherein females exhibit higher basal and inducible expression levels of TLR7, TLR9, IRF5, and IFN-*α* (166, 167). The sex differential expression of these pathways confers greater pro-inflammatory responses in peripheral blood mononuclear cells (PBMCs), neutrophils, and macrophages in males, whereas higher anti-inflammatory and cytokine signaling for type I IFN responses are seen in females (168, 169). Further, studies in rodents have shown that expression of signaling molecules associated with antiviral sensing and immunity (Myd88, IRF7, IFN-*β*, IFNAR1, JAK2, and STAT3) as well as antiviral protein Mx is higher in females compared to males (169). These dimorphic effects are posited to be mediated by gonadal hormones, with possible androgen- and estrogen-specific response elements driving different effector cells’ signaling and expression.

Despite these findings, studies directly looking at the sex differential contribution to viral susceptibility and disease outcome in humans are complicated by various behavioral and environmental differences associated with biological sex as well as gender. Several of the previously discussed viruses show preferential responses between females *versus* males, though whether biological differences are the cause of these observations is more difficult to tease out. Studies show that males have higher relative incidence of more serious illness and susceptibility to VZV and HSV-1, which may be explained by the aforementioned weakened immune response and pro-inflammatory cytokine profile (170, 171). However, interestingly, epidemiological studies show that females infected with Dengue virus in endemic areas have the same susceptibility to infection though exhibit more severe symptoms, such as hemorrhagic fever, compared to male counterparts (172). Females with Merkel cell carcinomas also have higher prevalence of MCPyV-positive tumors than male patients (173, 174). Additionally, HSV-2 shows a higher prevalence in females compared to males in humans (175, 176). These observations may appear to contradict immunological findings that females show a greater anti-inflammatory signature as well as an enhanced innate and adaptive immune profile compared to males. However, particularly in human studies, direct correlations of biological sex and viral susceptibility and disease outcome not only have to take into account sex hormones and chromosomal/genetic differences, they must also consider the differential effects that arise as a result of behaviors associated with gender and host environment, which may have direct consequences of increasing risk and susceptibility to certain viral pathogens. Murine studies have attempted to control for these confounding factors, although findings do not directly translate to humans. For example, increased progesterone levels are theorized to reduce immune-protective effects and therefore increase HSV-2 susceptibility in females. Female mice that underwent ovariectomy and had estradiol hormone injected showed reduced pathology compared to counterparts injected with progesterone or placebo (177). However, HSV-2 infection is increased in *ex vivo* human endometrial epithelial cells treated with estradiol (178). These divergent discoveries highlight the immense difficulty of using biological sex as a method of predicting viral susceptibility as well as disease outcome, although knowledge of sexual preferences of pathogens can be utilized to focus clinical efforts to provide better care to at-risk populations.

### Cutaneous Microbiome

The skin is home to a highly diverse collection of commensal bacteria, fungi and viruses that form the cutaneous microbiome. The makeup of these colonizers varies across individuals, skin compartments (*e.g.* hair follicle *versus* sebaceous gland), body location (*e.g.* axillary *versus* facial skin), and even age (179–181). This diversity is mirrored in the varying relationships between host skin and commensal microbiota, ranging from opportunistic to mutualistic interactions. For example, the *Cutibacteria* family (formerly known as *Propionibacteria)* of bacteria is a major component of normal skin flora that colonizes preferentially to skin sites that are rich in sebaceous glands. The presence of cutibacteria has been observed to impart protective benefits to the host in common skin pathologies including atopic dermatitis and psoriasis (182, 183). Conversely, *Cutibacterium acnes* often causes opportunistic infections and is a common etiologic agent in diseases such as acne vulgaris (184). These disparate consequences imply a necessity for the skin to maintain a healthy balance between itself and its surrounding microbiome. Furthermore, the predisposition for viral infection in populations with dysbiosis, such as those with atopic dermatitis, proposes the question of how microbial interactions influence skin responses to viral challenges (185).

Recent studies have begun to identify various antimicrobial roles of skin microbiota. Skin bacterial commensal *Staphylococcus epidermidis* was observed to produce peptides called bacteriocins that have direct antimicrobial properties against *Staphylococcus aureus* and Group A *Streptococcus* (186). Additionally, *S. epidermidis* was noted to augment the antimicrobial actions of cathelicidin LL-37 (187). *C. acnes* is also reported to secrete bacteriocins with bactericidal properties toward other cutibacteria (188). This work indicates that commensal bacteria actively participate in maintaining cutaneous microbial homeostasis; however, there is a current lack of understanding of antifungal and antiviral contributions from the cutaneous resident microbiota, including fungi and viruses.

Evidence of how the skin microbiome directly influences cutaneous antiviral immunity is also limited, although studies in patients with primary immunodeficiency, such as dedicator of cytokinesis 8 (DOCK8) deficiency who have altered cutaneous microbiomes compared to healthy patients, reveal that changes in the cutaneous virome lead to increased colonization of DNA viruses like HPVs, HSVs, polyomaviruses, and MCV (189). Inferences can additionally be drawn from studies in other barrier organs and their commensal microbiome. For instance, germ-free mice, *i.e.* lacking intestinal commensal microbiota, were observed to be more susceptible to influenza A virus, coxsackie B virus, Friend leukemia virus, and murine cytomegalovirus (190, 191). In the respiratory epithelium, *S. epidermidis* produced an extracellular matrix-binding protein that exhibited anti-influenza activity (192). Further, probiotic colonization of resident *Corynebacteria* improved resistance to respiratory syncytial virus (193). At the vaginal surface, lack of *Lactobacillus* bacteria, a dominant colonizer of the vaginal mucosa, led to increased susceptibility of HSV-2 due to abrogated IFN-*γ* signaling (194). Together, these findings suggest that commensal microbiota contribute directly to antiviral immunity *via* secretion of antiviral effectors and through enhancement of host immune signaling at their resident sites.

## Conclusion

The skin is an active immune organ with immune capabilities that are constantly challenged by friendly commensal and pathogenic microorganisms. Consequently, it has evolved effective defense strategies to combat a wide range of threats, ranging from overpopulation of opportunistic commensal bacteria to pathogenic viruses. Particularly in the scenario of viral infection, the skin’s complex multi-layered defense strategies, even within the innate immune system alone, are highlighted as different viruses’ attempt to hijack and suppress various aspects of its immune machinery. Given the severity of primary infections to many cutaneously introduced viruses, early antiviral responses are critical in the attempt to prevent further viral propagation and to allow time for adaptive immune responses to take effect. Recent advances in understanding specific viral targets of innate immunity begin to provide opportunities for further exploration into bolstering areas of vulnerability, including weaknesses that arise throughout age and between females and males. Additionally, insights to antiviral contributions from the commensal microbiome obtained from studies in other barrier organs suggest potential for future study in the skin.

## Author Contributions

VL and AM contributed to conception and design of the review. VL and AP performed initial literature search and wrote the first draft of the manuscript. AM supervised all aspects of the review and manuscript writing and is the corresponding author. All authors contributed to the article and approved the submitted version.

## Funding

VL is supported by the Burroughs-Wellcome and Poindexter Medical Student Research Fellowships. AM is supported by R01AI139207 and received a Duke Physician-Scientist Strong Start Award. AM is also supported by a Silab company partnership. The funding sources did not have any control over the content nor results of the review. The funders had no role in study design, data collection and analysis, decision to publish, or preparation of the manuscript.

## Conflict of Interest

AM consults for Silab and is on the scientific evaluation committee of the LEO Foundation and receives honoraria. AM’s spouse is employed by Precision BioSiences and holds stock and stock options. AA received the Pfizer Independent Grant for Learning and Change and has consulted for Henkel.

The remaining authors declare that the research was conducted in the absence of any commercial or financial relationships that could be construed as a potential conflict of interest.
